# Network pharmacology-based analysis of potential mechanisms of myocardial ischemia-reperfusion injury by total salvianolic acid injection

**DOI:** 10.3389/fphar.2023.1202718

**Published:** 2023-08-23

**Authors:** Nan Li, Xufang Gu, Fanqi Liu, Yao Zhang, Yanjun Sun, Shengwei Gao, Baohe Wang, Chen Zhang

**Affiliations:** ^1^ Tianjin University of Chinese Medicine, Tianjin, China; ^2^ The Second Affiliated Hospital of Tianjin University of Traditional Chinese Medicine, Tianjin, China; ^3^ The First Affiliated Hospital of Tianjin University of Traditional Chinese Medicine, Tianjin, China

**Keywords:** network pharmacology, myocardial ischemia-reperfusion injury, TSI, MI/RI, review

## Abstract

In this review, we investigated the potential mechanism of Total Salvianolic Acid Injection (TSI) in protecting against myocardial ischemia reperfusion injury (MI/RI). To achieve this, we predicted the component targets of TSI using Pharmmapper and identified the disease targets of MI/RI through GeneCards, DisGenNET, and OMIM databases. We constructed protein-protein interaction networks by analyzing the overlapping targets and performed functional enrichment analyses using Gene Ontology and Kyoto Encyclopedia of Genes and Genomes. Our analysis yielded 90 targets, which were implicated in the potential therapeutic effects of TSI on MI/RI. Seven critical signaling pathways significantly contributed to TSI’s protective effects, namely, PI3K signaling, JAK-STAT signaling, Calcium signaling, HIF-1 signaling, Nuclear receptor signaling, Cell Cycle, and Apoptosis. Subsequently, we conducted a comprehensive literature review of these seven key signaling pathways to gain further insights into their role in the TSI-mediated treatment of MI/RI. By establishing these connections, our study lays a solid foundation for future research endeavours to elucidate the molecular mechanisms through which TSI exerts its beneficial effects on MI/RI.

## Introduction

Ischemic heart disease persists as a prominent causative factor of mortality in cardiovascular disorders, encompassing both coronary heart disease and ischemic heart failure (Wong, 2014; Li et al., 2020). The clinical practice employs an array of pharmacological interventions to enhance coronary blood flow, encompassing anticoagulants, antiplatelet aggregation agents, nitroglycerin, and thrombolytics. Moreover, medical procedures like angioplasty or percutaneous coronary revascularization are employed to alleviate the impediments and reestablish the graceful flow of life-giving blood (Nakano et al., 2022).

However, the very act of restoring blood flow (reperfusion) post-ischemia unwittingly contributes to an exacerbation of damage at the hallowed site of myocardial ischemia, leading to a discernible escalation in the infarct area and a subsequent, consequential impact on myocardial function. This intricate phenomenon, widely recognized as myocardial ischemia reperfusion injury (MI/RI), bequeaths its tribulations upon the afflicted cardiac tissues (Li et al., 2014).

*Salvia miltiorrhiza*, a herb renowned for its traditional use in treating cardiovascular diseases like coronary heart disease, atherosclerosis, myocardial infarction, and angina pectoris, belongs to the class of herbs known to stimulate blood circulation and alleviate blood stasis (Wang et al., 2022). Notably, a study demonstrated that post-ischemic reperfusion recovery in left ventricular developed pressure was significantly improved, and contracture was reduced in hearts treated with *Salvia miltiorrhiza* compared to untreated hearts (Zhou and Ruigrok, 1990). The therapeutic effects of *Salvia miltiorrhiza* on MIRI are attributed to its active components, including tanshinone IIA, cryptotanshinone, and luteolin, which effectively target vascular endothelial growth factor A, interleukin-6, and AKT1. These active components act through vital regulatory pathways, namely, the PI3K/Akt signaling pathway, HIF-1 signaling pathway, and interleukin-17 signaling pathway (Jiang, 2021). Moreover, using a network pharmacology approach, Salvianolic Acid A, another critical constituent of Danshen, has been meticulously investigated for its therapeutic potential in treating myocardial infarction (MI). The study identified several therapeutic targets, such as SRC, CTNNB3, PIK1CA, AKT1, RELA, EGFR, FYN, ITGB8, MAPK1, and NFKB29, and their gene expressions were validated in an H9C2 cell OGD/R model using RT-qPCR. Notably, the PI3K/Akt signaling pathway demonstrated significant association with MI, indicating its critical role in the therapeutic mechanism of Salvianolic Acid A (Huang et al., 2022). Another study highlighted the protective effects of Salvianolic Acid A against I/R-induced myocardial damage by reducing necrosis and apoptosis in isolated rat hearts and cardiomyocytes through the PI3K/Akt signaling pathway, consequently increasing the Bcl-2/Bax ratio (Pan et al., 2011). Furthermore, Wang’s research revealed that tanshinone primarily acts early after ischemic injury by inhibiting intracellular calcium and cell adhesion pathways. At the same time, Salvianolic Acid A chiefly exerts its effects by down-regulating apoptosis (Wang et al., 2011). Moreover, Salvianolic Acid A inhibited myocardial I/R injury by suppressing inflammatory responses and inhibiting high mobility group box 1 expression via the PI3K/AKT pathway (Liu et al., 2020). Overall, these findings underscore the scientific basis and logical mechanisms behind *Salvia miltiorrhiza* and its active components’ therapeutic efficacy in treating cardiovascular diseases and protecting against myocardial ischemia reperfusion injury.

Total Salvianolic Acid Injection (TSI) is a water-soluble *Salvia miltiorrhiza* substance. The Institute of Drug Research, the Chinese Academy of Medical Sciences, and Tianjin Tianshili Pharmaceutical Co. Created it, and studies have shown it to be helpful in MI/RI (Si et al., 2016; Huang et al., 2019).

Due to the rapid progress in bioinformatics and pharmacology, network pharmacology has emerged as a cost-effective approach for conducting pharmacological research for drug development. This methodology involves investigating the intricate relationships among compounds, proteins/genes, and diseases from a holistic network standpoint. It has garnered significant attention in drug discovery as it facilitates the exploration of potential “compound-protein/gene-disease” pathways (Zhang et al., 2019).

In this study, we aimed to investigate the underlying mechanism of TSI against MI/RI and provide valuable insights for treating MI/RI. We employed a network pharmacology approach to comprehensively analyze the interactions between TSI and its potential targets to systematically understand the molecular pathways underlying its anti-MI/RI effects to achieve this goal. Insights gained from this study will pave the way for further experimental studies and ultimately aid in developing new therapeutic strategies for treating myocardial infarction and reperfusion injury.

## Network pharmacology

**FIGURE 1:**
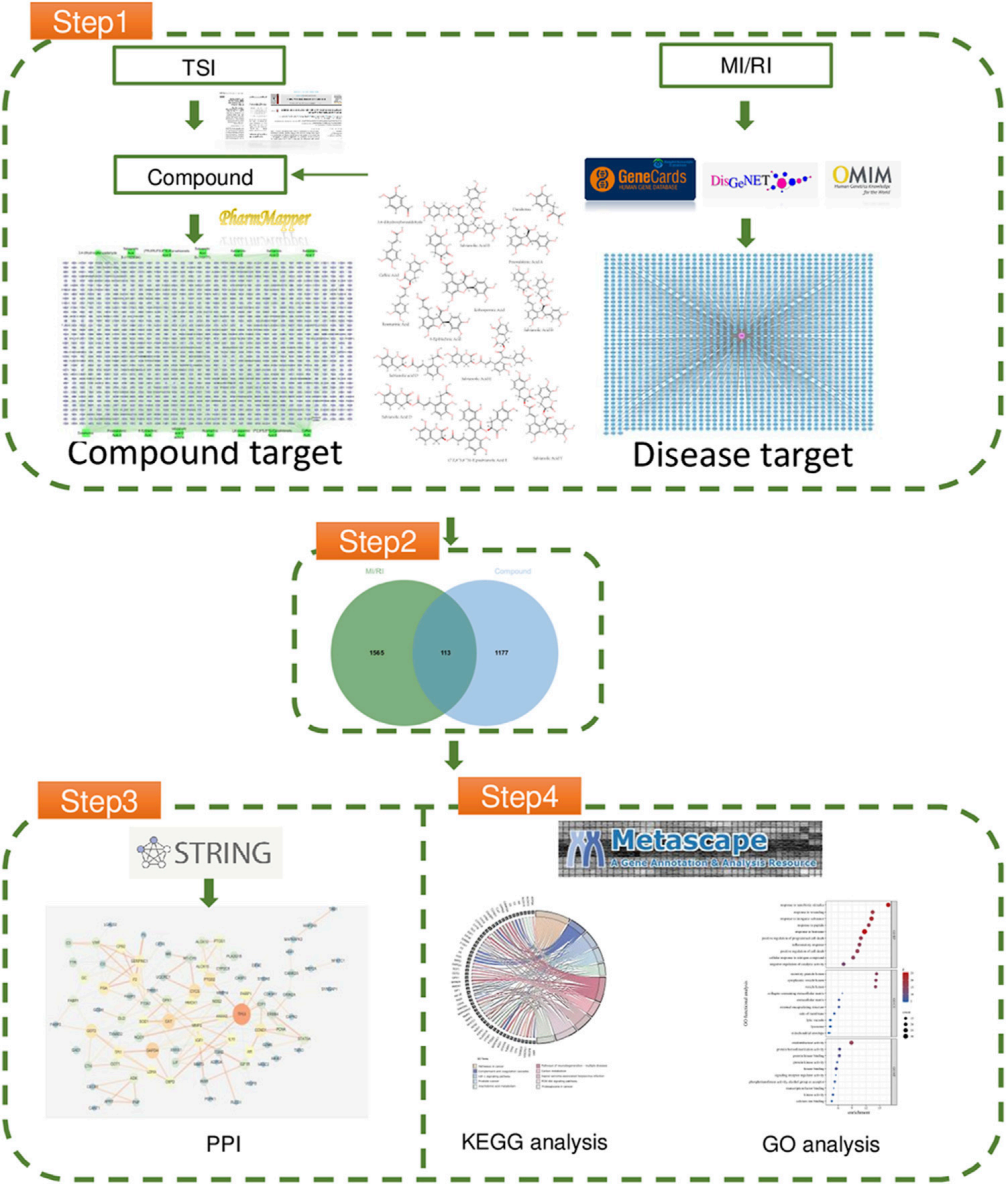
The flowchart of network pharmacology.

The particular steps consist of the following (Li et al., 2020): using literature to determine the composition of TSI (Wong, 2014), using the database to determine the relevant targets of MI/RI (Nakano et al., 2022); performing protein interaction on the intersection targets; and (Li et al., 2014) performing Gene Ontology and Kyoto Encyclopedia of Genes and Genomes functional enrichment analyses on the targets of protein interaction results. For the workflow diagram, see Figure 1.

### Screening of potential targets

The literature was consulted to determine the compound of TSI; three articles described the compound of TSI(Si et al., 2016; Jie-feng et al., 2018; Huang et al., 2019). 14 compounds were obtained in all. The SDF-formatted of these compounds were retrieved using Pubchem, and the chemical structures not present in PubChem were manually animated and exported using Swiss Target Prediction (see Supplementary Figure S1) (Daina et al., 2019). The SDF-formatted compounds were imported into Pharm Mapper (Version 2017) to match possible targets (see Supplementary Figure S2). Using “myocardial ischemia reperfusion injury” as a search term in GeneCards (Stelzer et al., 2016), DisGenNET (Piñero et al., 2020), and OMIM (Amberger et al., 2019) to identify MI/RI disease targets (see Supplementary Figure S3). Standardizing the target names with the UniProt ID and removing duplicate targets resulted in 1,289 compound targets and 1,678 disease targets. There were 113 intersecting targets in total (see Supplementary Figure S4).

### Constructing the protein-protein interaction network

Protein-protein interaction (PPI) is a physical contact formed when two or more protein molecules interact via electrostatic forces, hydrogen bonds, *etc.* Intercellular contacts, cell cycle processes, signal transduction, and metabolic pathways are biological processes in which PPI plays a significant role. It is essential to investigate the PPI of TSI with MI/RI for pharmaceutical efficacy. The intersection of disease targets and drug targets. We imported the targets obtained in step one into String (Version 11.5) (Szklarczyk et al., 2015) to generate the nodes and lines of the action network of PPI with confidence set to 0.90, exported the data to Cytoscape (Shannon et al., 2003), deleted the free proteins that did not form a network, and obtained 90 nodes with 198 edges (see Supplementary Figure S5).

### Gene ontology analysis

The PPI targets are imported into Metascape for Gene Ontology (GO) enrichment analysis. The GOs of the most abundantly enriched genes are rated, and the top 10 are selected (see Supplementary Figure S6). The top-ranked biological processes associated with cell death include positive regulation of cell death and positive regulation of programmed cell death. They are also associated with oxidoreductase activity, protein kinase binding, phosphotransferase activity, calcium ion (Ca^2+^) binding, transcription factor binding, and signaling receptor regulator activity.

### Kyoto encyclopedia of genes and genomes pathway analysis

Kyoto Encyclopedia of Genes and Genomes (KEGG) is an online database on genomic, enzymatic pathways, and biochemistry developed in Japan. To better understand the probable mechanism of TSI on MI/RI, we imported the PPI-acquired targets into Metascape for KEGG enrichment analysis to determine the pathways with which the targets are connected. Finally, 119 pathways were enriched, ranked according to logP, with the top 10 KEGG pathways displayed in Supplementary Figure S7 The signals of the top 10 pathways were retrieved using the KEGG PATHWAY Database (https://www.genome.jp/kegg), as shown in Table 1. Signals that appeared more than twice were considered significant signals, and we reviewed the significant signals.

**TABLE 1:** The signals of the top 10 KEGG pathway.

*KEGG pathway*	*Signling*	*KEGG pathway*	*Signling*
Pathways in cancer	ERK signaling	Carbon metabolism	Carbohydrate metabolism
	**PI3K signaling**		Energy metabolism
	Other RAS signaling		Amino acid metabolism
	WNT signaling	PI3K-Akt signaling pathway	PI3K-Akt signaling
	NOTCH signaling	Kaposi sarcoma-associated herpesvirus infection	MAPK signaling
	HH signaling		**PI3K signaling**
	TGFB signaling		**JAK-STAT signaling**
	**JAK-STAT signaling**		**Calcium signaling**
	**Calcium signaling**		TLR signaling
	**HIF-1 signaling**		IFN signaling
	KEAP1-NRF2 signaling		TNF signaling
	**Nuclear receptor signaling**		Chemokine signaling
	**Cell cycle**		MHC presentation
	**Apoptosis**		Autophagy
	Telomerase activity		**Cell cycle**
Complement and coagulation cascades	Complement cascade		**Apoptosis**
	Coagulation cascade	Arachidonic acid metabolism	-
	Combined network	Pathways of neurodegeneration - multiple diseases	-
Prostate cancer	**PI3K signaling**	Proteoglycans in cancer	-
	**Nuclear receptor signaling**	HIF-1 signaling pathway	**HIF-1 signaling**
	**Cell cycle**		

Bold indicates important signal.

## Review of potential mechanisms of TSI on MI/RI

### PI3K signaling

**FIGURE 2:**
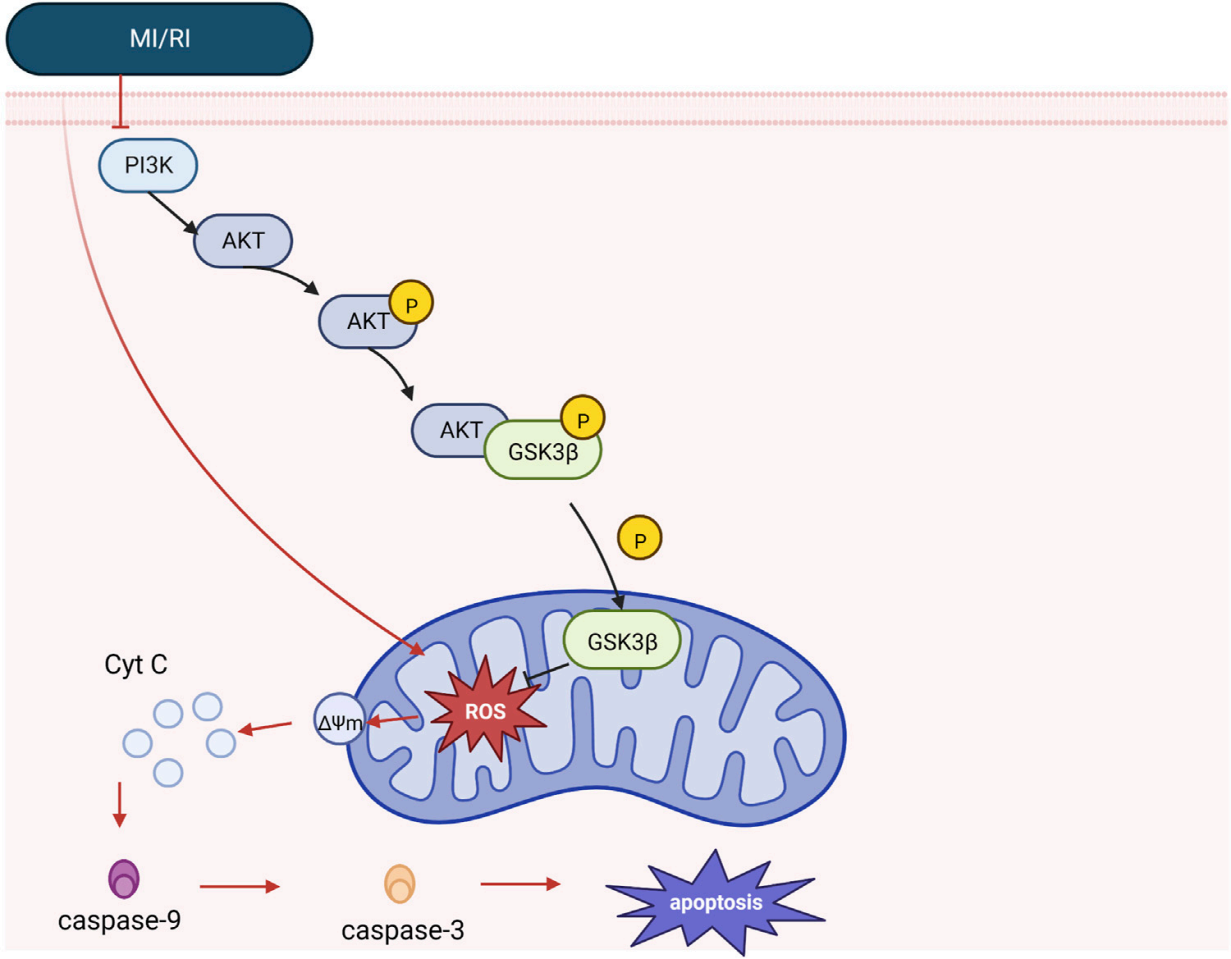
Mechanistic of PI3K signaling. Under physiology, mitochondrial integrity is maintained by PI3K/Akt signaling, and Akt phosphorylates GSK3β. However, in the case of MI/RI, PI3K/Akt signaling was inhibited, while MI/RI caused excessive ROS production in cell mitochondria, increased oxidative stress, altered mitochondrial membrane potential, released cyt c and caspase-9, promoted the formation of apoptotic vesicles, increased caspase-3 release, and promoted the apoptotic cascade response (see apoptosis mechanism diagram for details).

Phosphatidylinositol-3-kinase (PI3K) and the downstream target serine/threonine kinase (Akt) constitute a signaling pathway with critical roles in various physiological processes that promote cell survival in response to noxious external stimuli (Li et al., 2021). In addition, PI3K/Akt pathway activation attenuates mitochondrial-mediated apoptosis and maintains mitochondrial integrity through phosphorylated molecules (e.g., Bcl2 family and Glycogen synthase kinase-3 beta) (Wang et al., 2018a). In addition, hypoxic reoxygenation dramatically downregulates p-PI3K and p-AKT expression, whereas hypoxic reoxygenation pretreatment with Piperine boosted p-PI3K and p-AKT expression (Li et al., 2021). MI/RI overproduces reactive oxygen species (ROS), increases oxidative stress, alters Mitochondrial membrane potential, and releases mitochondrial cytochrome c (cyt c) into the cytoplasm, ultimately activating caspase-3 in the apoptotic pathway. Ginsenoside Rd was found to reduce intracellular ROS production in cardiomyocytes through PI3K/Akt/Glycogen synthase kinase-3 beta (GSK3β) signaling inhibits mitochondria-dependent apoptosis during MI/RI (Wang et al., 2013). Using *in vitro* and *in vivo* experiments, Peng Yu also revealed that Elabela could prevent cardiac fibrosis, increase mitochondrial function, and reduce apoptosis and oxidative stress via the PI3K/AKT signaling pathway in MI/RI (Yu et al., 2020). See Figure 2. Mechanistic of PI3K signaling.

### JAK-STAT signaling

**FIGURE 3:**
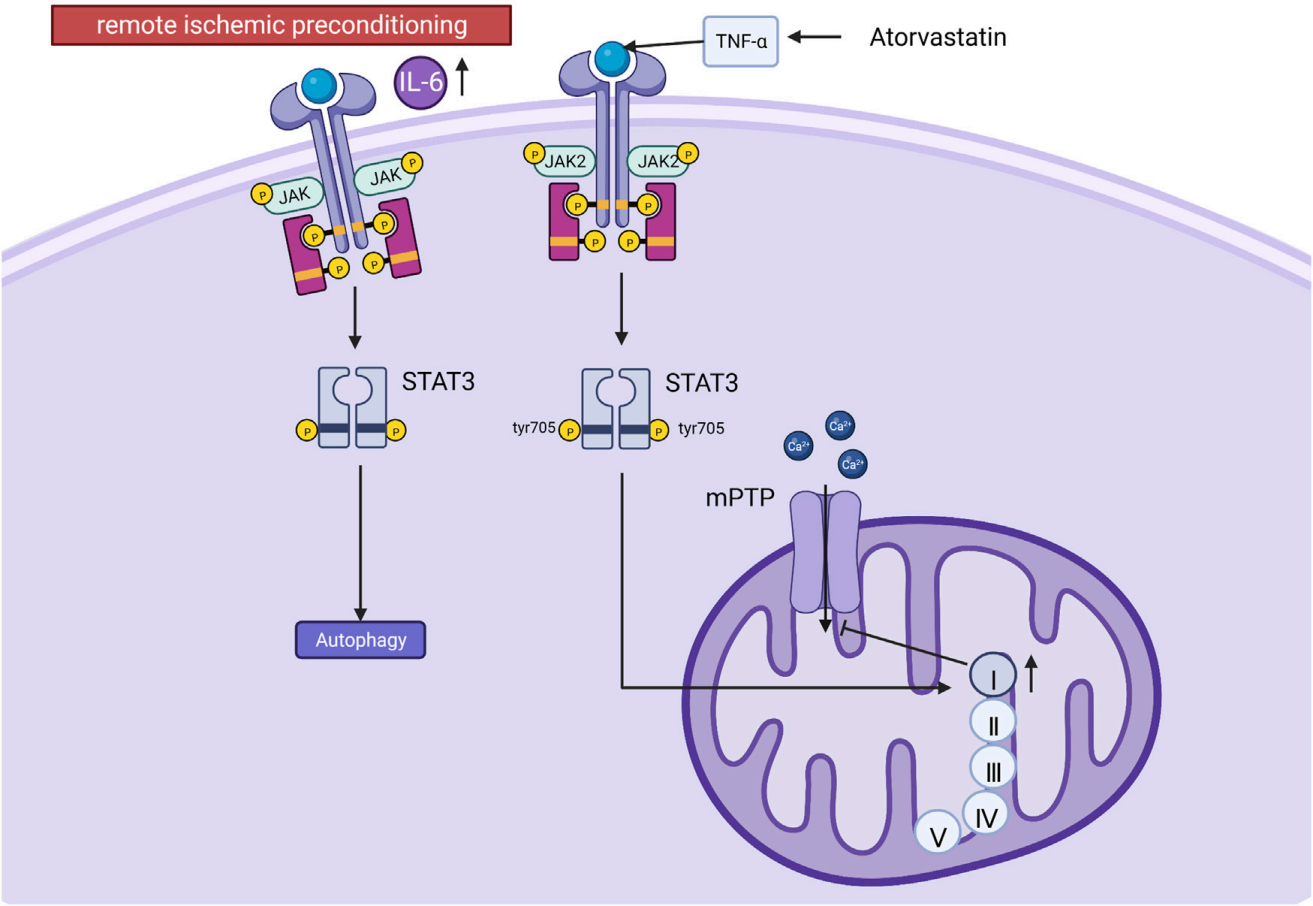
Mechanism of JAK/STAT signaling. Post-ischemic treatment increases STAT3 phosphorylation at tyrosine 705 (tyr 705), promotes mitochondrial complex I respiration, maintains mPTP sensitivity to opening, and inhibits mPTP storage capacity for calcium. Remote ischemic preconditioning increases plasma IL-6, regulates autophagy via the JAK/STAT3 pathway, and protects against MI/RI. Atorvastatin activates JAK2 and STAT3 via TNF-α.

The JAK-STAT signaling pathway is a chain of interactions between proteins within the cell. This signaling pathway transmits information from extracellular peptide signaling directly to the nucleus through transmembrane receptors that activate genes via transcription. JAK-STAT signaling has three key components: Janus kinases (JAKs), signal transducer and activator of transcription proteins (STATs), and receptors (which bind the chemical signals) (Aaronson and Horvath, 2002). JAK contains four proteins: JAK1, JAK2, JAK3 and TYK2; STAT contains seven proteins: STAT1, STAT2, STAT3, STAT4, STAT5A, STAT5B, STAT6. JAK is rapidly recruited to the receptor and activated after receiving signals from upstream receptor molecules. The activated JAK catalyzes the complexion phosphorylation of the receptor, which ultimately leads to gene transcription. JAK1 and JAK2 are activated by MI/RI, which activates STAT1 (promotes apoptosis) and STAT3 (protects cardiomyocytes) (Stephanou, 2004; Boengler et al., 2008). It was shown that post-ischemic treatment could further increase STAT3 phosphorylation at tyrosine 705 and reduce the area of myocardial infarction in MI/RI pigs. STAT3 phosphorylation at tyrosine705 better promotes respiration in mitochondrial complex I (probably because activated STAT3 promotes docking of protein kinases to their targets and indirectly affects respiration) (Pfeffer et al., 1997). Since the electron flux of complex Ⅰ is essential for maintaining the sensitivity of the mitochondrial permeability transition pore (mPTP) to open (Fontaine et al., 1998). Therefore, it can indirectly inhibit the ability of the mPTP to store Ca^2+^, and the above effect will be eliminated by AG490 (JAK2/STAT3 pathway inhibitor) (Heusch et al., 2011). Similarly, results from rat experiments and cardiomyocyte experiments suggest that melatonin reduces myocardial ischemia-induced mitochondrial-induced oxidative damage by activating the JAK2/STAT3 signaling pathway. Previous studies have reported that cardioprotective strategies such as ischemic preconditioning and post-adaptation can upregulate autophagy (Gurusamy et al., 2009; Huang et al., 2011). Muntasir Billah showed that remote ischemic preconditioning increases plasma circulating interleukin 6(IL-6) and regulates cardioprotective autophagy via the JAK/STAT3 pathway, thereby protecting against MI/RI (Billah et al., 2020). In addition, atorvastatin enhanced the recovery of contractility after hypoxia-reoxygenation via tumor necrosis factor-α (TNF-α) activation and phosphorylation of JAK2 and STAT3. It is shown that activation of TNF-α is required for JAK2 and STAT3 phosphorylation (Lemoine et al., 2013). See Figure 3. Mechanism of JAK/STAT signaling.

### Calcium signaling

**FIGURE 4:**
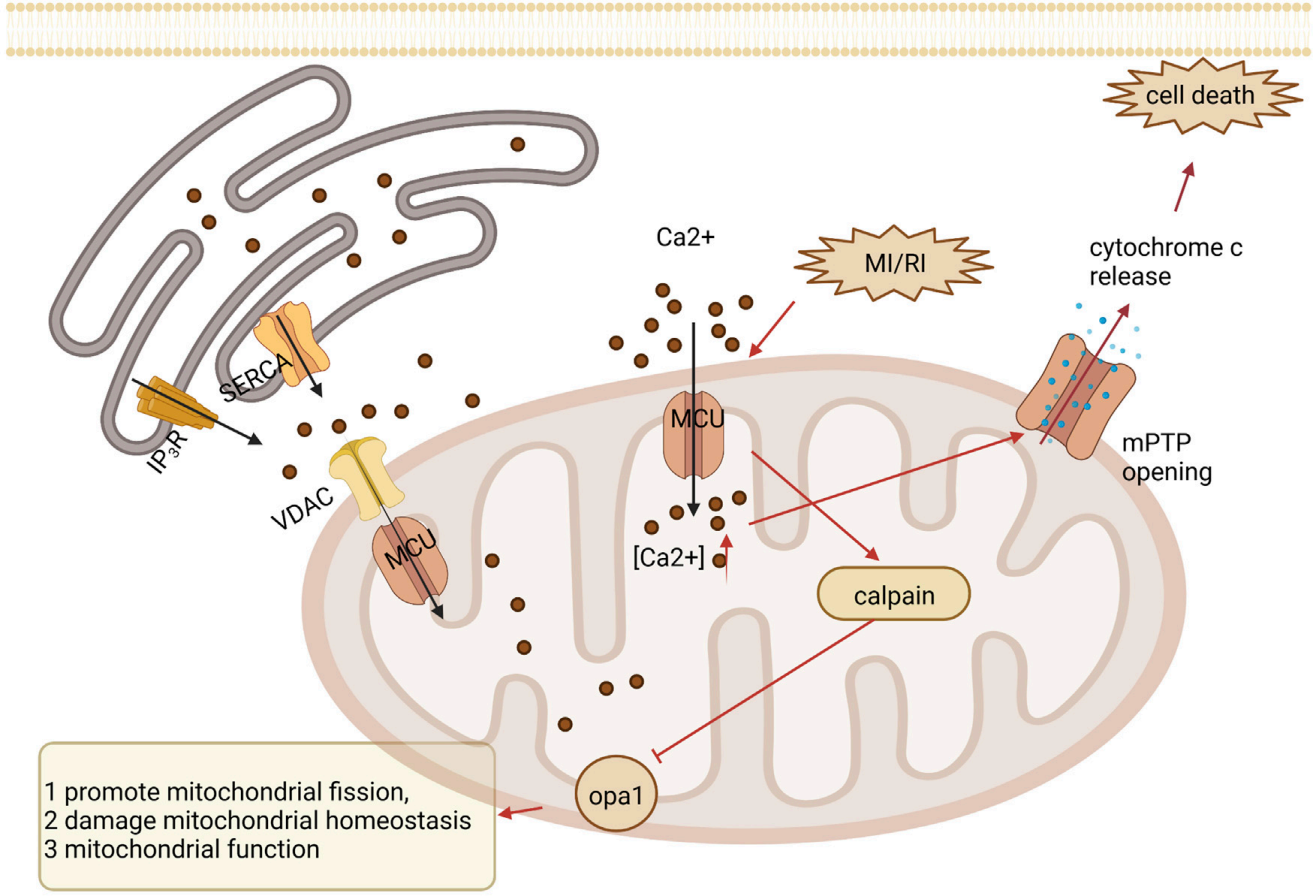
Mechanisms of calcium signaling. Under MI/RI, MCU causes mitochondrial Ca2+ overload, opening of the mPTP, and cell death. During myocardial reperfusion, MCU-induced mitochondrial calcium overload activates calpain, which inhibits optic atrophy type 1 (OPA1), thereby inhibiting mitochondrial fusion and mitochondrial autophagy and promoting mitochondrial division, leading to impaired mitochondrial morphology and function, resulting in cardiomyocyte apoptosis.

Calcium signaling uses Ca^2+^ to communicate and drive intracellular processes, often as a step in signal transduction. Ca^2+^ is a critical second messenger in regulating mitochondrial tasks and a key link in the coupling of excitation metabolism and excitation contraction in the heart. The inner mitochondrial membrane has a complex system of Ca^2+^ uptake and efflux channels and transporters that decode cytoplasmic Ca^2+^ signals and store Ca^2+^ in the mitochondrial matrix (Dedkova and Blatter, 2013). Intracellular and mitochondrial Ca^2+^ overload occurs in the myocardium during ischemia and is exacerbated during reperfusion. In addition, the mitochondrial calcium uniporter (MCU), located in the inner mitochondrial membrane, is the essential unidirectional channel responsible for Ca^2+^ influx into mitochondria, and the MCU regulates mitochondrial Ca^2+^ homeostasis (Murphy and Steenbergen, 2008; Williams et al., 2015). Under cardiac MI/RI stress, MCU causes mitochondrial Ca^2+^ overload, the opening of the mPTP, and cell death (Villanueva et al., 2017). And the upregulation of MCU may increase intracytoplasmic Ca^2+^ through sarcoplasmic reticulum-mitochondria communication. In addition, μ-calpain (calpain-1) and m-calpain (calpain-2) are the major isoforms of calpain expressed in cardiac myocytes. During myocardial reperfusion, mitochondrial Ca^2+^ overload caused by MCU activates calpain, which causes MI/RI through different mechanisms, including increased membrane fragility and mitochondrial dysfunction (Inserte et al., 2012; Smith and Schnellmann, 2012). Moreover, optic atrophy type 1 resides on the mitochondrial membrane and regulates mitochondrial fusion. Activated calpain suppresses optic atrophy type 1, inhibiting mitochondrial fusion and autophagy and promoting mitochondrial division, decreasing mitochondrial morphology and function and cardiomyocyte apoptosis (Guan et al., 2019). See Figure 4. Mechanisms of calcium signaling.

### Nuclear receptor signaling

**FIGURE 5:**
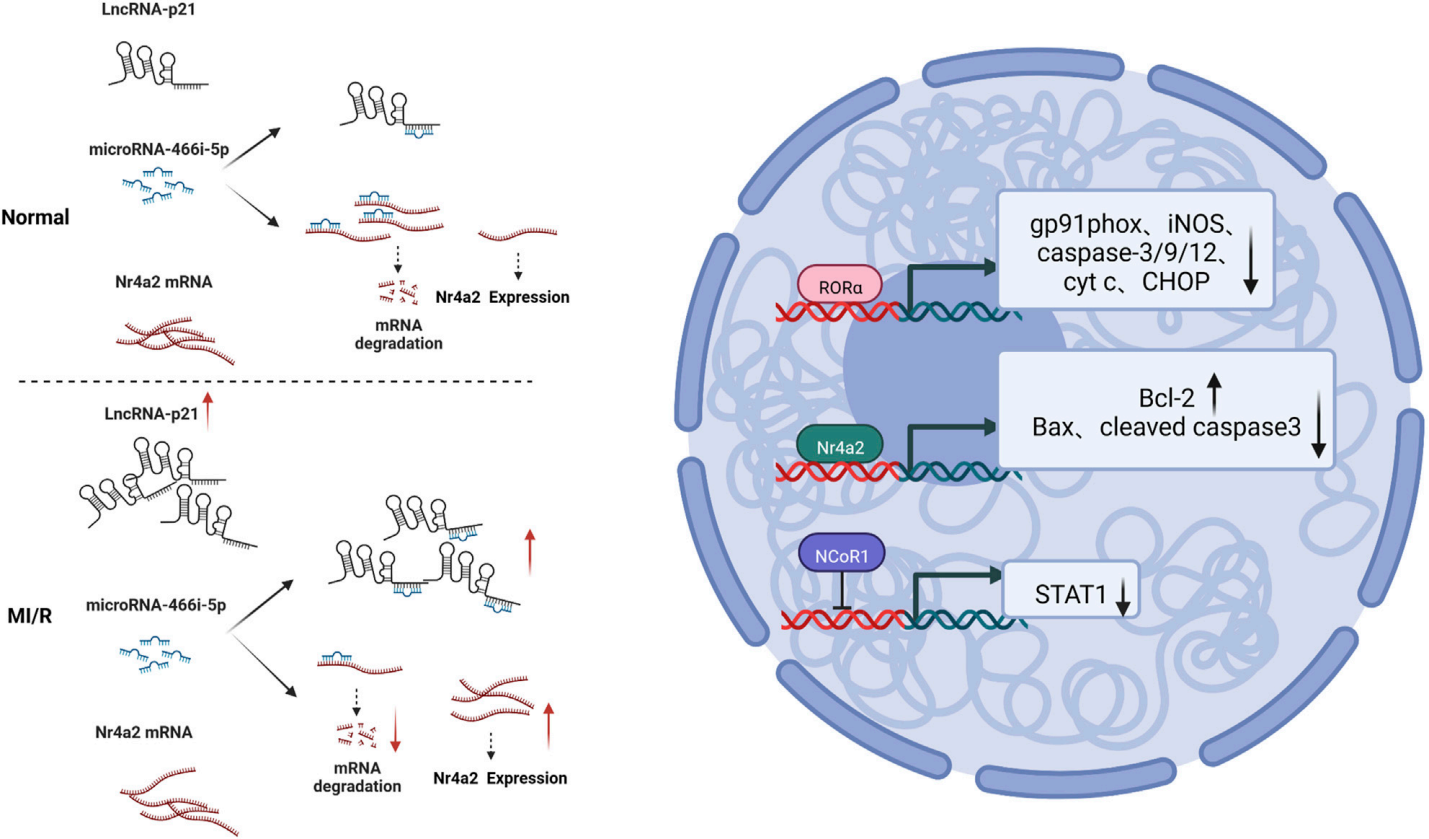
Mechanism of nuclear receptor signaling. MI/RI upregulated LnRNA-21, which caused more miRNA-466i-5p to bind competitively to it, resulting in less binding of Nr4a2 mRNA to miRNA-466i-5p and, relatively, more Nr4a2 mRNA expression, and increased Bcl-2 expression and downregulated Bax and cleaved caspase 3 expression; RORα inhibited expression of gp91phox, iNOS, caspase-3/9/12, cyt c and CHOP; NCoR1 inhibited STAT1 expression.

Nuclear receptors are a class of proteins that sense other molecules, such as steroids, thyroid hormones, *etc.* Nuclear receptors bind to DNA that regulates the expression of adjacent genes, so these receptors are called transcription factors (Rm, 1988; Olefsky, 2001). Nuclear receptor subfamily 4 group A member 2 (Nr4a2) is a nuclear receptor. It was discovered that lncRNA-p21 is a competing endogenous RNA (ceRNA) for microRNA-466i-5p, so inhibition of lncRNA-p21 promotes the expression of microRNA-466i-5p, thereby inhibiting the expression of Nr4a2 and ultimately inhibiting MI/RI led to infarct size and improved cardiac function. It was also found that inhibition of LincRNA-p21 increased the expression of anti-apoptotic factor b-cell lymphoma-2 (Bcl-2), downregulated the expression of Bcl-2-associated X protein (Bax), and cleaved caspase-3. This indicates that the anti-apoptotic effect of the lncRNA-p21/miR-466i-5p/Nr4a2 pathway on myocardial damage can be achieved via this mechanism (Zhai et al., 2022). Retinoid-related orphan receptors (RORs) regulate circadian rhythms and mediate melatonin’s effects; ROR and ROR isoforms have been identified in the adult mouse heart. Endogenous ROR can ameliorate mitochondrial dysfunction (mitochondrial swelling, caspase-9 activation, and cytochrome c release), downregulate ER stress (caspase-12 activation and CHOP expression), and inhibit apoptosis (TUNEL-positive cells) by inhibiting pro-oxidant enzyme (gp91phox and iNOS) expression and caspase-3 activation) as well as enhancing the recovery of autophagy after MI/RI (He et al., 2016). The nuclear receptor co-repressor 1 (NCoR1), also known as thyroid hormone- and retinoic-acid-receptor-associated co-repressor 1 (TRAC-1), includes nuclear receptor co-activators and co-repressors that regulate a variety of physiological processes including cell survival and death signaling. The investigators observed that NCoR1 expression in myocardial tissue was downregulated in MI/RI. That cardiomyocyte-specific NCoR1 knockdown exacerbated acute reperfusion-induced cardiac injury through mitochondria-mediated apoptosis (manifested by enlargement of myocardial infarction, reduction of left ventricular contractility, reduction of surviving myocardium, and exacerbation of myocardial inflammatory edema). And cardiomyocyte-specific NCoR1 deficiency critically promotes activation of inflammatory pathways, and it is well known that intracellular activation of signal transducer and activator of transcription 1 (STAT1) is closely associated with abnormal expression of inflammatory cytokines (Wang et al., 2018b). Moreover, epigenomic and transcriptomic analysis data identified STAT1 as a direct transcriptional repressor target of NCoR1. This demonstrates that cardiomyocyte-expressed NCoR1 functions as a crucial cardioprotective factor against acute MI/RI by targeting the STAT1 pathway in the heart (Qin et al., 2022). See Figure 5. Mechanism of nuclear receptor signaling.

### HIF-1 signaling

**FIGURE 6:**
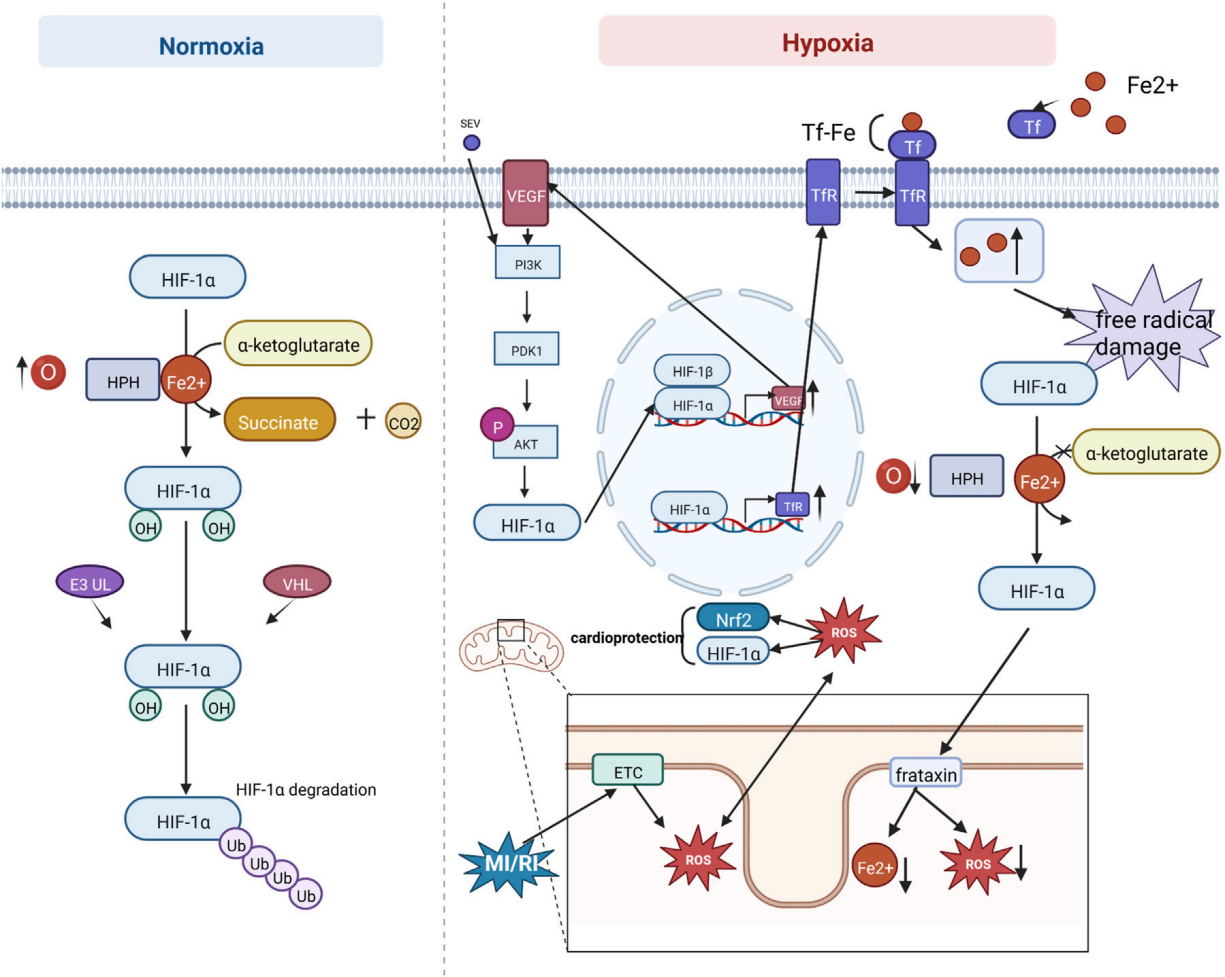
Mechanism of HIF-1 signaling. Under normoxic conditions, HIF-1α is degraded via the ubiquitin-protease pathway. During hypoxia, intracellular hypoxia inhibits HIF-1α activation by HIF-prolyl hydroxylases (HPHs), interacts with many coactivators, and regulates target gene expression. Sevoflurane (SEV) pretreatment can increase VEGF expression through the PI3K/AKT/HIF-1α signaling pathway. In addition, ROS produced by mitochondrial electron transport chain (ETC) can impair mitochondrial function to promote apoptosis, while stabilizing HIF-1α and the antioxidant transcription factor Nrf2 to protect cardiac function. HIF-1α activates upregulation of transferrin receptor (TfR) expression, which increases transferrin (Tf) binding iron uptake and increases intracellular iron levels, leading to iron catalytic free radical damage.

Hypoxia-inducible factor-1(HIF-1) is an oxygen-sensitive transcription factor. It consists of a beta subunit (also termedaryl hydrocarbon receptor nuclear translocator) and an alpha subunit. Under normoxic conditions, HIF-1α is degraded via the ubiquitin-protease pathway (Ke and Costa, 2006). During hypoxia, intracellular hypoxia inhibits the activation of HIF-1α by HIF-prolyl hydroxylases, also referred to as prolyl hydroxylase domain proteins, interacts with many coactivators, and regulates target gene expression. HIF-1α was found to regulate the expression level of the mitochondrial protein frataxin, and increased frataxin attenuated mitochondrial iron overload and ROS production, thereby maintaining mitochondrial membrane integrity and cellular activity (Nanayakkara et al., 2015; Chowdhury et al., 2018). In addition, sevoflurane pretreatment can increase the expression level of vascular endothelial growth factor (VEGF) by activating the Akt/HIF-1α/VEGF signaling pathway, increase angiogenesis in the focal area of MI/RI, improve myocardial hypoxia, and thus protect the heart (Dong et al., 2019). In addition, ROS produced by the mitochondrial electron transport chain has a bidirectional regulatory effect, as they can impair mitochondrial function and promote apoptosis while stabilizing HIF-1α and the antioxidant transcription factor (nuclear factor erythroid 2-related factor 2) Nrf2 to protect cardiac function (Cadenas, 2018). However, HIF-1α has a detrimental side. Studies have shown that HIF-1α activates the upregulation of transferrin receptor expression, increasing transferrin binding iron uptake and intracellular iron levels, leading to iron-catalyzed free radical damage (Tang et al., 2008). See Figure 6. Mechanism of HIF-1 signaling.

### Cell Cycle

**FIGURE 7:**
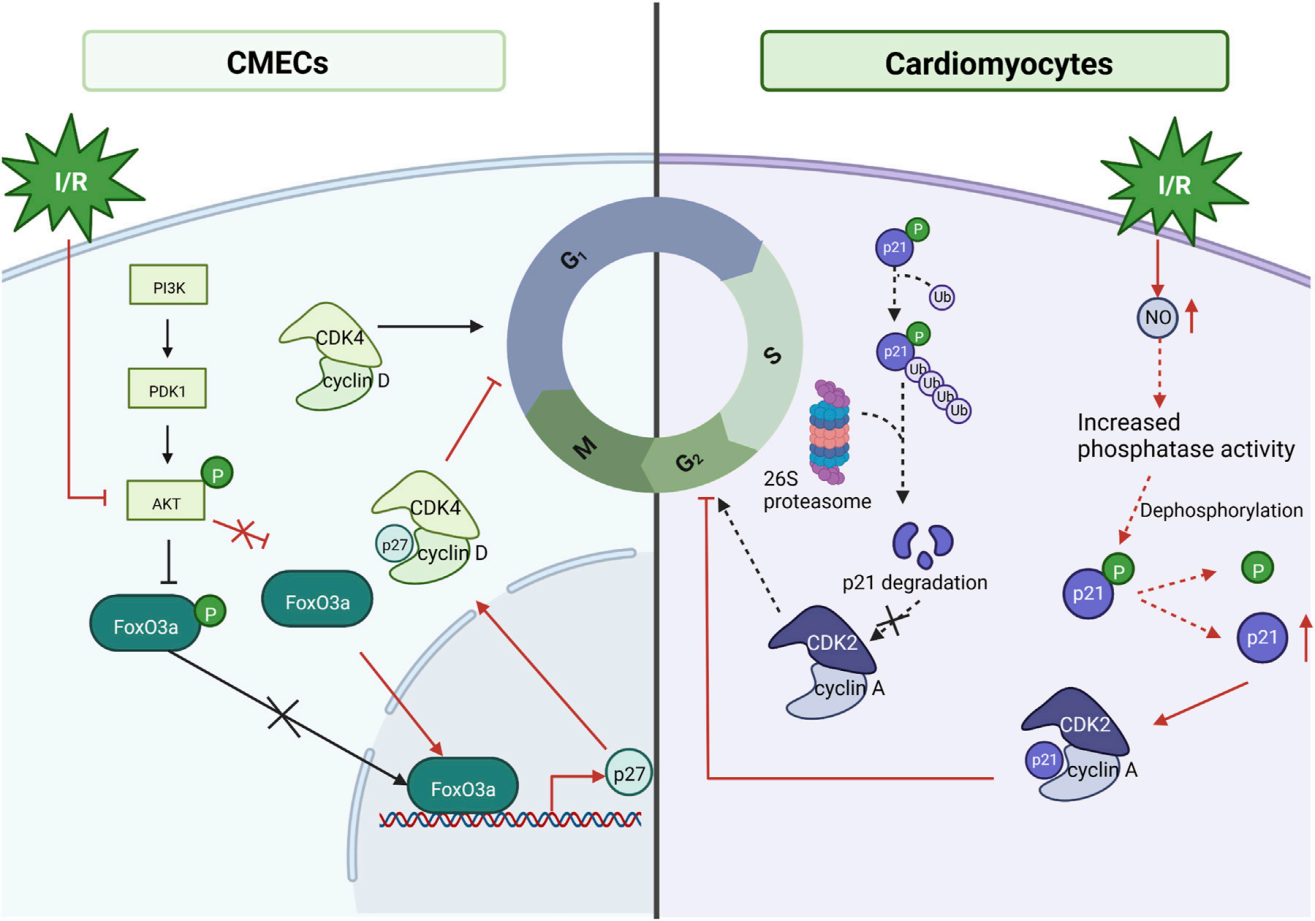
Mechanism of Cell cycle. Under MI/RI, cardiac microvascular endothelial cells (CMECs) are impaired, FoxO3a pathway is activated, AKT is inactivated, and AKT fails to inhibit FoxO3a, allowing FoxO3a to control the cell cycle by activating p27 ^Kip1^ expression, inhibiting CDK4, and arresting cells in G1 phase. MI/RI induces NO production in cardiomyocytes, inhibits CDK2 activity through p21^cip/waf1^ accumulation to inhibit CDK2 activity and upregulate cyclin A/CDK2 activity thereby inhibiting apoptosis.

The cell cycle is a series of processes that lead to dividing a single cell into two daughter cells. In cardiac myocytes, dysregulation of cell cycle control has been linked to MI/RI (Stefka et al., 2000; Haley et al., 2008). The proliferative cell cycle requires the transmission of mitogenic signals to the cell cycle proteins; cyclin and cyclin-dependent-kinase (CDK) binding catalyzes the activity of CDKs, which regulate cell progression through the various cell cycle phases by regulating the activity of critical substrates that allow cells to enter each cell cycle (Perteguer et al., 2013). In mammals, there are two CDK inhibitors: the INK4 family, which is exclusive to CDK4/6 and contains p15, p16, p18, and p19; and the CIP/KIP family, which inhibits all G1/S CDKs and includes p21, p27, and p57 (MacLellan, 2010). FoxO3a (also known as FKHRL-1) is a member of the FoxO subfamily of forkhead transcription factors, a key target of the PI3K/AKT pathway that is phosphorylated by AKT and inactivated by phosphorylated FoxO3a, a process associated with metabolism, differentiation, proliferation, cell cycle and apoptosis (Huang and Tindall, 2006; He et al., 2013). The experimental results showed that in the case of I/R, cardiac microvascular endothelial cells were impaired, the FoxO3a pathway was activated, AKT was inactivated (mentioned above as having a protective effect against cardiac I/R), and FoxO3a controlled the cell cycle by activating p27 expression, inhibiting CDK4 and arresting the cells in G1 phase (Medema et al., 2000). In addition, it has been indicated that MI/RI induces nitric oxide (NO) production in cardiomyocytes, endogenous NO is involved in cell cycle regulation, inhibits CDK2 activity through p21 accumulation, and upregulates cell cycle protein A/CDK2 activity, thereby inhibiting apoptosis (Maejima et al., 2003). Although the mechanism by which NO increases p21 was not shown in this study, Melina R. Kibbe showed that NO prevents p21 degradation through the ubiquitin-proteasome pathway associated with increased protein tyrosine and serine/threonine phosphatase activity in vascular smooth muscle cells (Kibbe et al., 2000). Therefore, it is speculated that in cardiomyocytes in NO also, p21 is increased by this mechanism, but further experimental verification is needed (Haley et al., 2008). It is evident that cell cycle arrest is a protective mechanism for MI/RI and that MI/RI causes apoptosis while turning on a cytoprotective mechanism (Kibbe et al., 2000). See Figure 7. Mechanism of Cell cycle.

### Apoptosis

**FIGURE 8:**
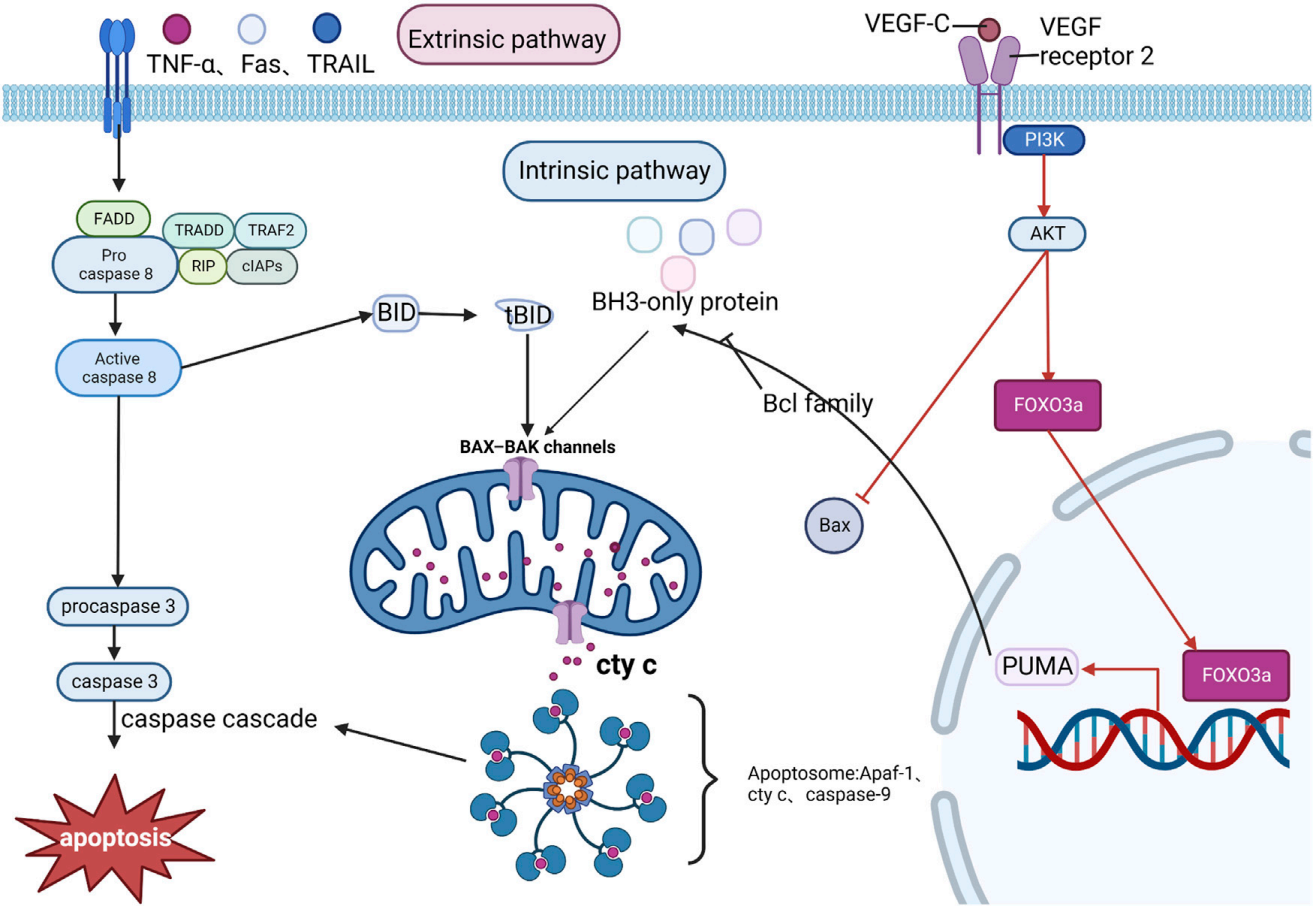
Mechanism of Apoptosis. The exogenous pathway, activated by pro-inflammatory ligands, is aided by FADD, which continuously accumulates Procaspase 8 and catalyzes the formation of caspase 8, which activates other caspase families to form a caspase cascade reaction that promotes apoptosis. In the endogenous pathway, caspase 8 hydrolyzes BID to form tBID, which promotes the release of cyt c and the assembly of apoptotic vesicles Apaf-1 and caspase-9, triggering the caspase cascade response. MI/RI can inhibit Bax expression through the PI3K/AKT pathway and exert cardiomyocyte protection, in addition to the PI3K/AKT pathway promotes FOXO3a to regulate PUMA at the transcriptional level, thereby promoting apoptosis.

MI/R aggravates myocardial injury and leads to apoptosis of cardiomyocytes, which can lead to a decrease in cardiac function. The apoptosis process is divided into two phases: the initiation phase and the effector phase. The initiation phase is divided into two pathways: the intrinsic pathway and extrinsic pathway. The extrinsic pathway is activated by pro-inflammatory ligands (e.g., TNF-α, Fas, and TRAIL) via plasma membrane receptors, and with the assistance of FADD (Fas-associated death domain protein), the TNF receptor family continuously collects procaspase-8 collected in the cytoplasm and a high density of procaspase-8 autocatalyzes the formation of caspase-8 which will trigger a caspase cascade reaction. In the intrinsic pathway, caspase-8 can mediate the hydrolysis of the BH3-only protein BID to form a truncated BID (tBID), promoting the release of cyt c and the assembly of apoptotic vesicles Apaf-1 and caspase-9. The intrinsic pathway usually requires the activation of BH3-only proteins of the Bcl family. When BH3-only protein activation reaches a certain amount, it can overcome the inhibition of Bcl-2 family members and promote the assembly of granule outer membrane Bax-Bak oligomers, where mitochondrial outer membrane permeabilization occurs, allowing the release of pro-apoptotic factors such as cyt c, which enters as pre-apoptotic substances in the cytoplasm, which together with Apaf-1 and caspase-9 form the apoptosome and cause the caspase cascade reaction (Lovell et al., 2008; Taylor et al., 2008; Edlich et al., 2011). The pro-apoptotic protein Bax shuttles between cytoplasmic and granule outer membrane in healthy conditions. Under apoptotic conditions, VEGF-C (a pro-angiogenic protein) can activate the PI3K/AKT signaling pathway via VEGF receptor 2 and inhibit Bax expression and its translocation to the mitochondrial membrane, preventing mitochondrial outer membrane permeabilization and thus exerting a protective effect against MI/RI-induced and ROS-mediated cardiomyocyte apoptosis (Chen et al., 2016). p53-upregulated modulator of apoptosis (PUMA), also known as Bcl-2-binding component 3, belongs to the BH3-only protein family and is a pro-apoptotic protein that inhibits anti-apoptotic effects (Nakano and Vousden, 2001). FOXO3a can regulate PUMA at the transcriptional level (You et al., 2006). It was found that FOXO3a/PUMA signaling was activated in cardiomyocytes subjected to MI/R injury and that Dexmedetomidine pretreatment inhibited mitochondrial oxidative stress, thereby blocking this signaling to prevent apoptosis in cardiomyocytes (Yang et al., 2022). Xiangwei Lv showed that miRNA-346 overexpression could target and inhibit Bax expression, suppressing apoptosis and adequately protecting the myocardium from I/R. Therefore, miR-346/Bax may become a new mechanism and potential therapeutic target for preventing MI/R injury (Lv et al., 2020). See Figure 8. Mechanism of Apoptosis.

## Conclusion

In this study, we employed a network science approach to explore potential TSI and MI/RI targets. We performed functional enrichment analysis to elucidate the underlying mechanism of TSI’s effect on MI/RI. In addition, we extensively reviewed and discussed vital signaling pathways, including PI3K signaling, JAK-STAT signaling, calcium signaling, HIF-1 signaling, nuclear receptor signaling, cell cycle, and apoptosis, to lay a literature foundation for future studies on the mechanism of TSI in the treatment of MI/RI. However, it is essential to acknowledge that our study lacks experimental validation, and further investigation is required to confirm our findings. Specifically, measuring protein or mRNA expression levels of potential targets identified in our network analysis would provide crucial experimental evidence supporting our predictions. Ultimately develop more effective treatment strategies for MI/RI management.

## Abbreviations

Akt, serine/threonine kinase; Bax, Bcl-2-associated X protein; Bcl-2, b-cell lymphoma-2; CDK, cyclin-dependent-kinase; ceRNA, endogenous RNA; cyt c, cytochrome c; GO, Gene Ontology; GSK3β, Glycogen synthase kinase-3 beta; HIF-1, Hypoxia-inducible factor-1; IL-6, interleukin 6; JAKs, Janus kinases; KEGG, Kyoto Encyclopedia of Genes and Genomes; MCU, mitochondrial calcium uniporter; MI/RI, myocardial ischemia reperfusion injury; mPTP, mitochondrial permeability transition pore; NCoR1, nuclear receptor co-repressor 1; NO, nitric oxide; Nr4a2, Nuclear receptor subfamily 4 group A member 2; PI3K, Phosphatidylinositol-3-kinase; PPI, Protein-protein interaction; PUMA, p53-upregulated modulator of apoptosis; RORs, Retinoid-related orphan receptors; ROS, reactive oxygen species; STAT1, signal transducer and activator of transcription 1; STATs, signal transducer and activator of transcription proteins; tBID, truncated BID; TNF-α, tumor necrosis factor-α; TRAC-1, thyroid hormone- and retinoic-acid-receptor-associated co-repressor 1; TSI, Total Salvianolic Acid Injection; VEGF, vascular endothelial growth factor.
