# Supplementary figures and images for: Network pharmacology-based analysis of potential mechanisms of myocardial ischemia-reperfusion injury by total salvianolic acid injection
Source: Front Pharmacol. 2023 Aug 23;14:1202718. doi: 10.3389/fphar.2023.1202718 (PMC10482107; doi:10.3389/fphar.2023.1202718)

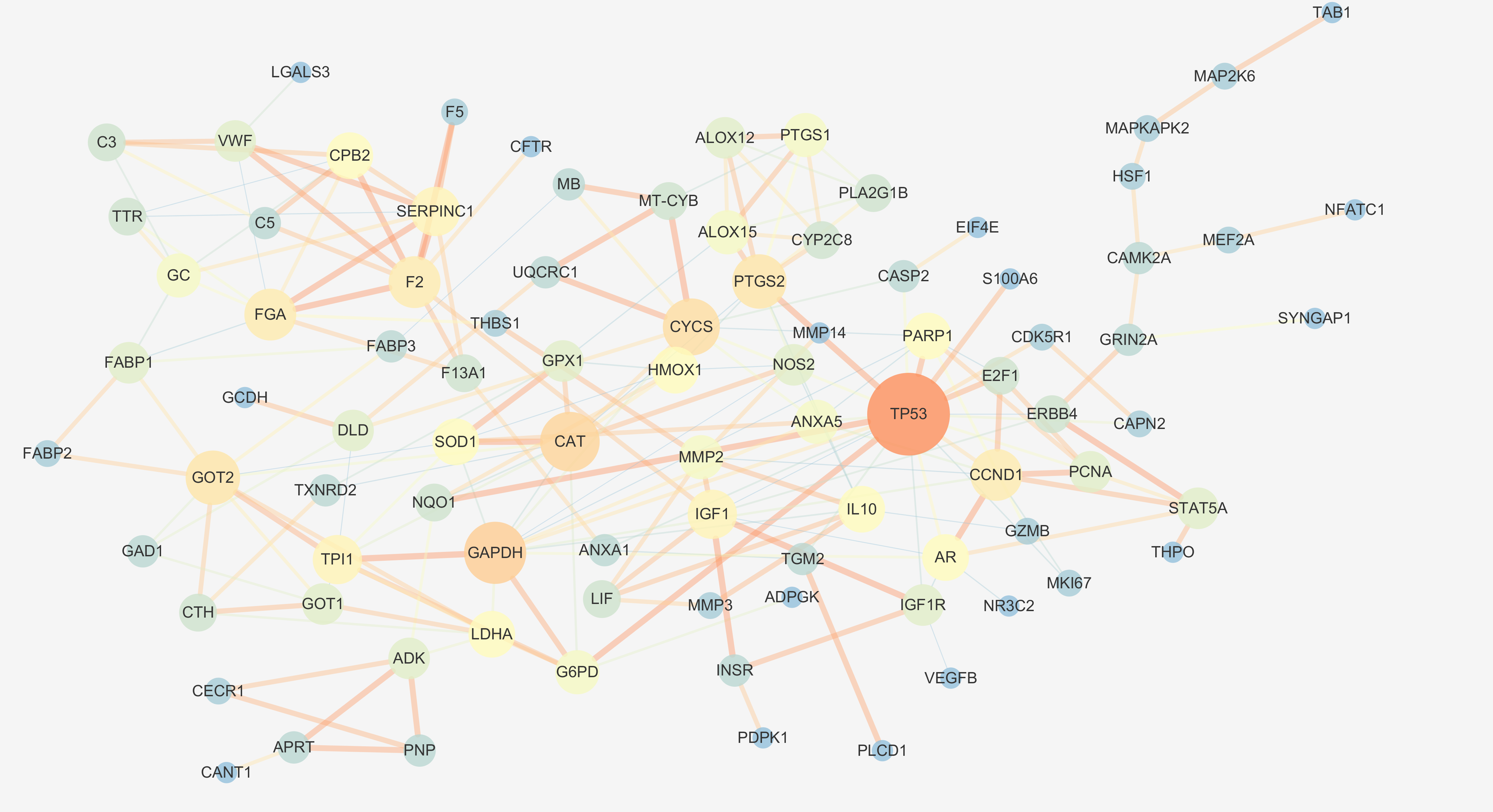

Supplement: Supplementary file 1 [file Image5.PNG]

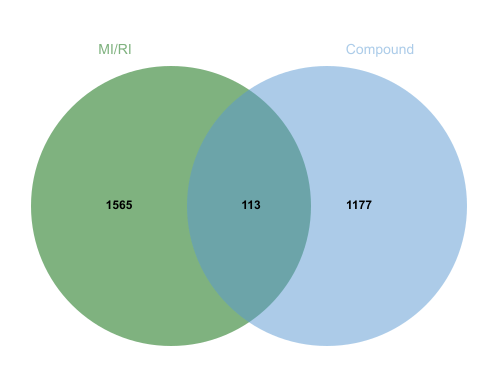

Supplement: Supplementary file 2 [file Image4.PNG]

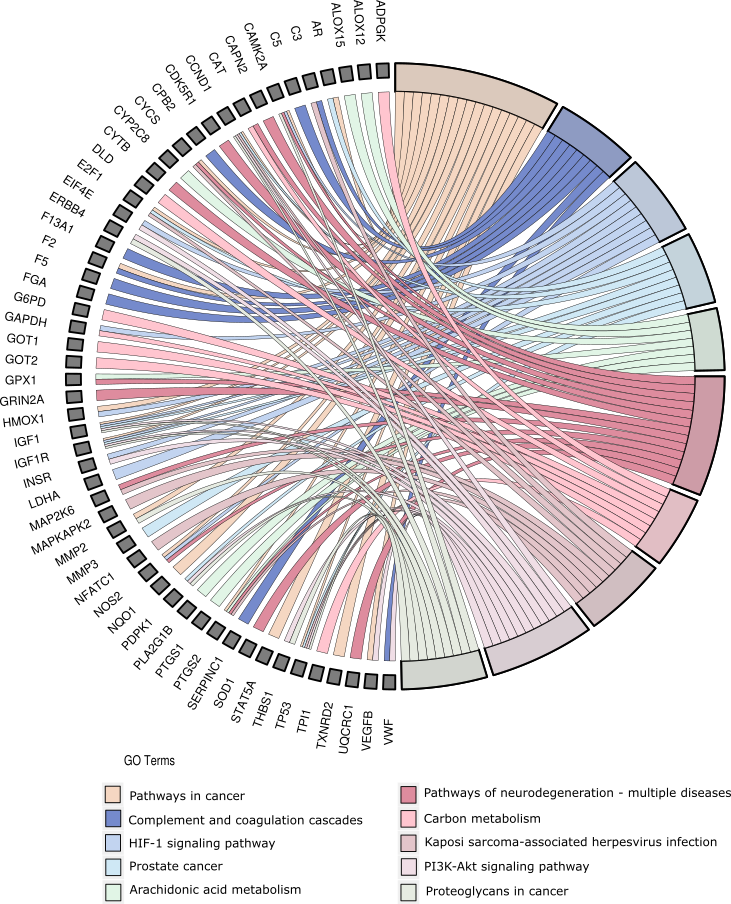

Supplement: Supplementary file 3 [file Image7.PNG]

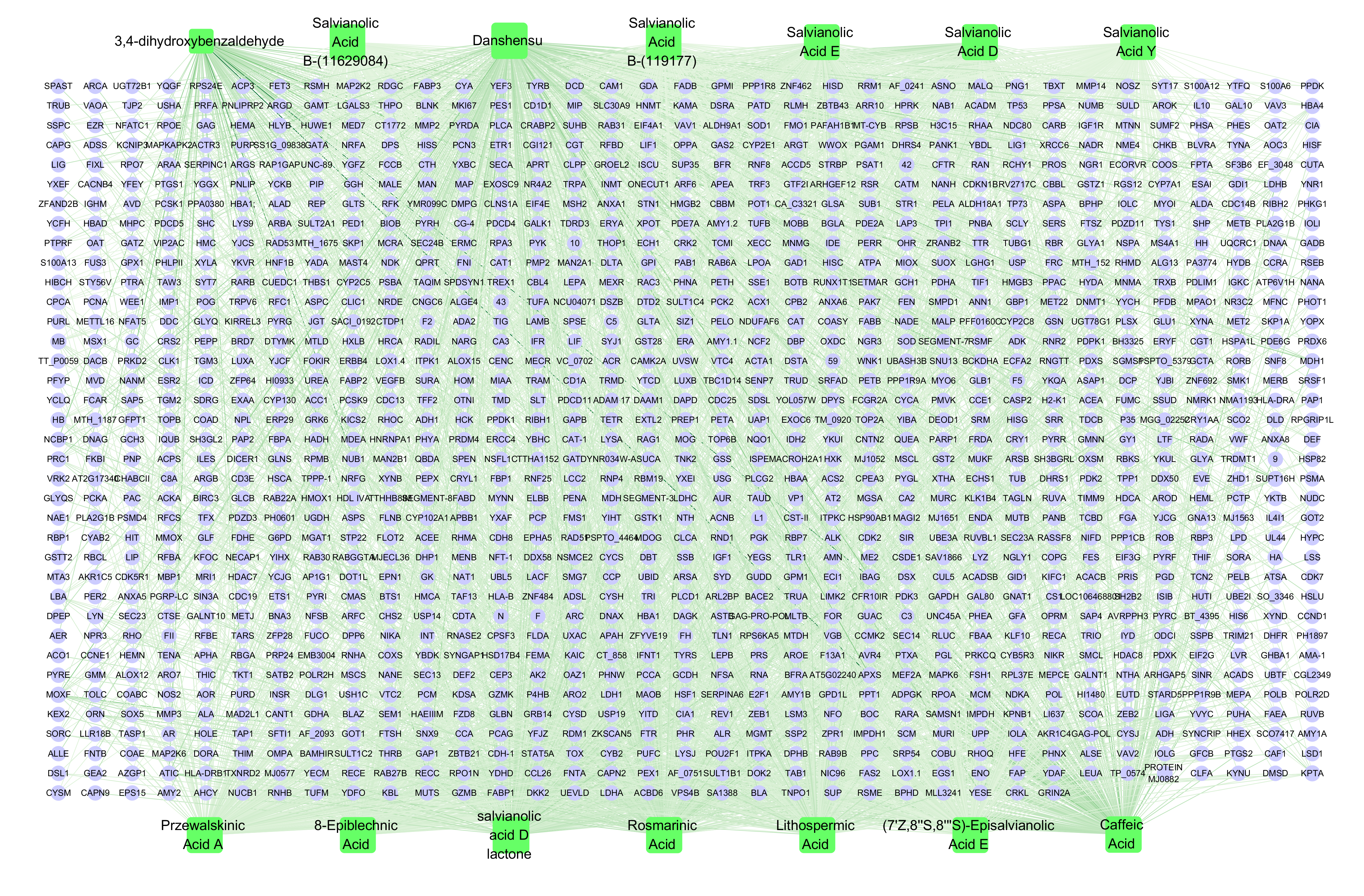

Supplement: Supplementary file 4 [file Image2.PNG]

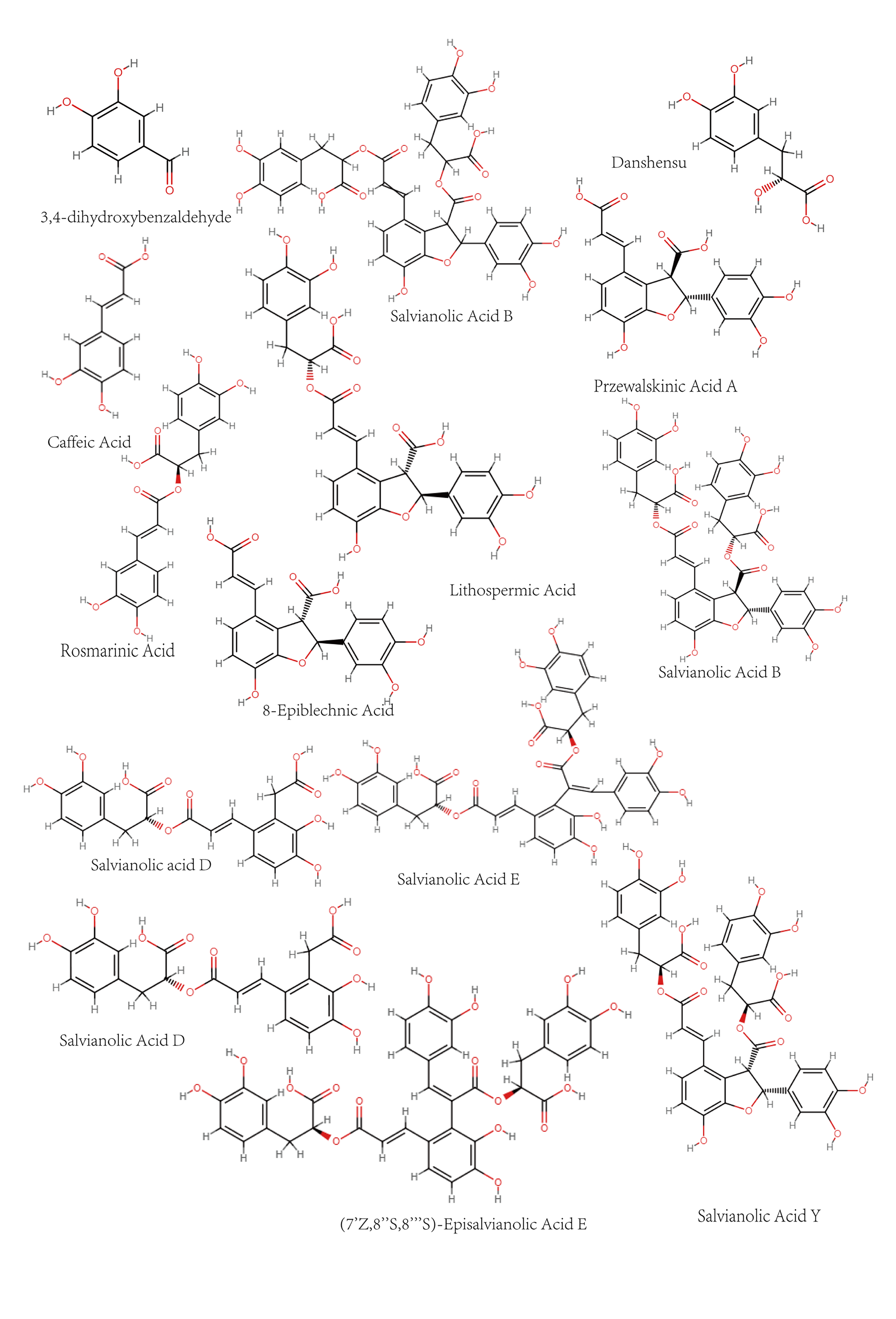

Supplement: Supplementary file 5 [file Image1.PNG]

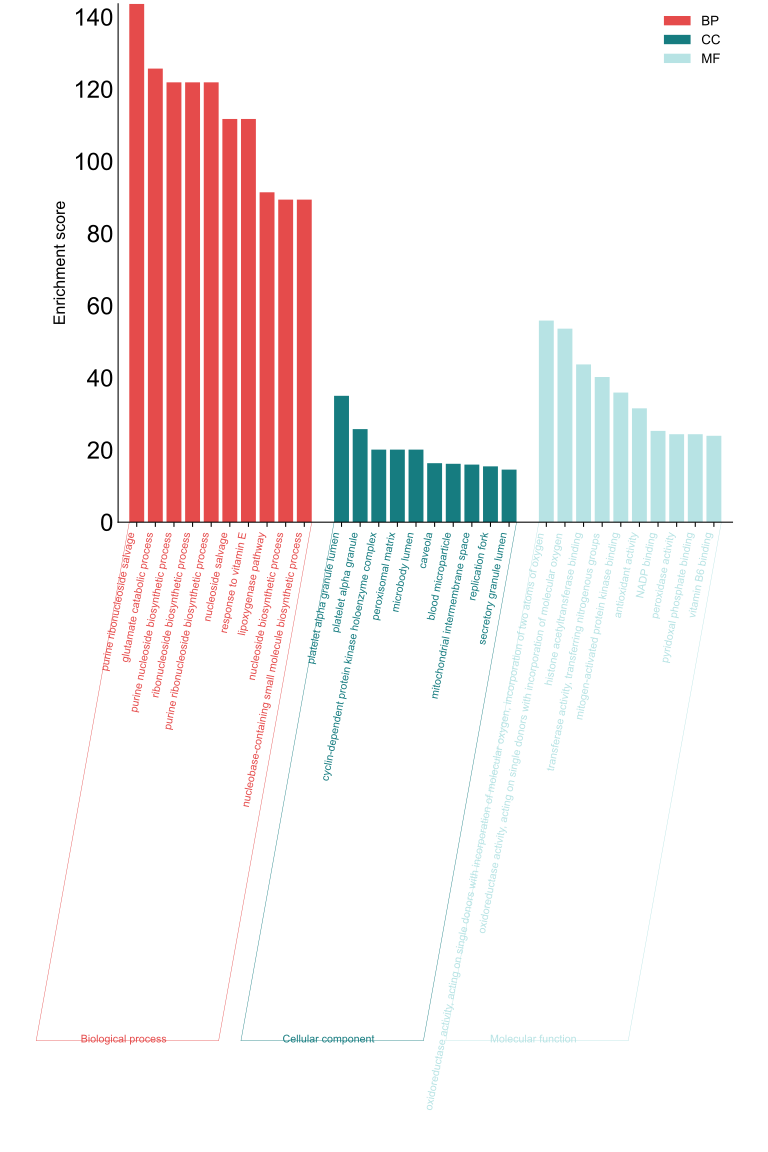

Supplement: Supplementary file 6 [file Image6.PNG]

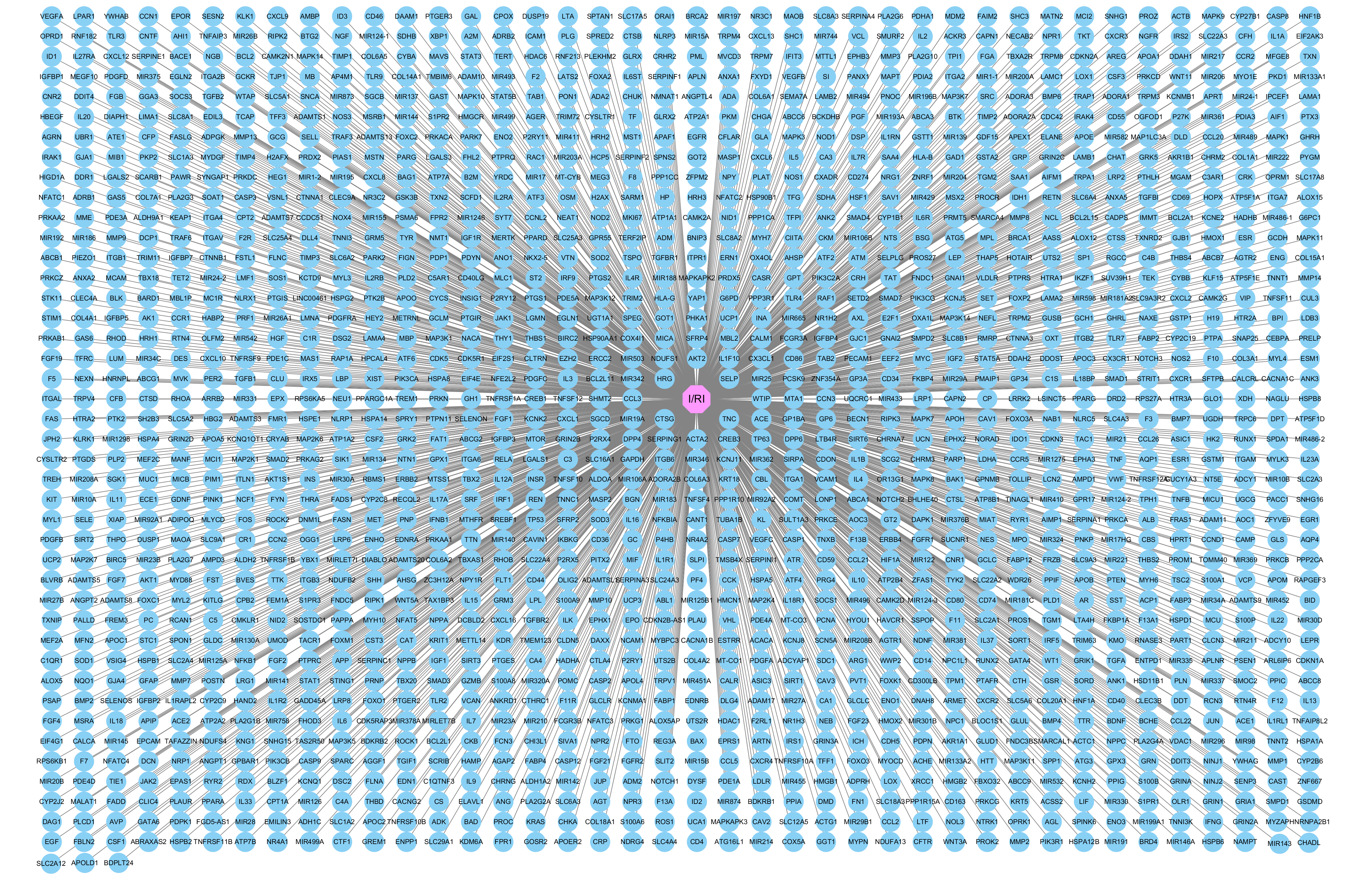

Supplement: Supplementary file 7 [file Image3.PNG]
